# Convergence in insulin resistance between very severely obese and lean women at the end of pregnancy

**DOI:** 10.1007/s00125-015-3708-3

**Published:** 2015-08-07

**Authors:** Shareen Forbes, Sarah M. Barr, Rebecca M. Reynolds, Scott Semple, Calum Gray, Ruth Andrew, Fiona C. Denison, Brian R. Walker, Jane E. Norman

**Affiliations:** Tommy’s Centre for Fetal and Maternal Health, MRC Centre for Reproductive Health, University of Edinburgh, Queen’s Medical Research Institute, Edinburgh, UK; Endocrinology Unit, University/BHF Centre for Cardiovascular Science, University of Edinburgh, Queen’s Medical Research Institute, 47 Little France Crescent, Edinburgh, EH16 4TJ UK; Clinical Research Imaging Centre, University of Edinburgh, Queen’s Medical Research Institute, Edinburgh, UK

**Keywords:** Insulin resistance, MRI, Pregnancy, Stable isotope studies, Very severe obesity

## Abstract

**Aims:**

Disrupted intermediary metabolism may contribute to the adverse pregnancy outcomes in women with very severe obesity. Our aim was to study metabolism in such pregnancies.

**Methods:**

We recruited a longitudinal cohort of very severely obese (*n* = 190) and lean (*n* = 118) glucose-tolerant women for anthropometric and metabolic measurements at early, mid and late gestation and postpartum. In case–control studies of very severely obese and lean women we measured glucose and glycerol turnover during low- and high-dose hyperinsulinaemic–euglycaemic clamps (HEC) at early and late pregnancy and in non-pregnant women (each *n* = 6–9) and body fat distribution by MRI in late pregnancy (*n* = 10/group).

**Results:**

Although greater glucose, insulin, NEFA and insulin resistance (HOMA-IR), and greater weight and % fat mass (FM) was observed in very severely obese vs lean participants, the degree of worsening was attenuated in the very severely obese individuals with advancing gestation, with no difference in triacylglycerol (TG) concentrations between very severely obese and lean women at term. Enhanced glycerol production was observed in early pregnancy only in very severely obese individuals, with similar intrahepatic FM in very severely obese vs lean women by late gestation. Offspring from obese mothers were heavier (*p* = 0.04).

**Conclusions/interpretation:**

Pregnancies complicated by obesity demonstrate attenuation in weight gain and insulin resistance compared with pregnancies in lean women. Increased glycerol production is confined to obese women in early pregnancy and obese and lean individuals have similar intrahepatic FM by term. When targeting maternal metabolism to treat adverse pregnancy outcomes, therapeutic intervention may be most effective applied early in pregnancy.

**Electronic supplementary material:**

The online version of this article (doi:10.1007/s00125-015-3708-3) contains peer-reviewed but unedited supplementary material, which is available to authorised users.

## Introduction

The prevalence of obesity is increasing in women of childbearing age [1, 2]. In the UK 20% of pregnant women are obese and 2% have very severe obesity (BMI ≥40 kg/m^2^) [3]. The adverse maternal consequences of obesity in pregnancy include Caesarean section, haemorrhage and infection [4–6] along with stillbirth and neonatal intensive care unit admission [7]. Children born to obese mothers are more likely to be large for gestational age (LGA) (and small for gestational age [SGA]) and have a greater lifetime risk of obesity [8–10], with health [11] and economic implications [12].

The mechanism by which maternal obesity causes harm to mother and child is unclear, and disrupted intermediary metabolism may play a major role [13]. In the non-pregnant state, obese individuals are insulin resistant with high circulating levels of metabolites and inflammatory markers, and a predisposition to hyperglycaemia [14, 15]. Pregnancy is an insulin-resistant state and the consequent mobilisation of fatty acids and glucose provide substrates for fetal growth [16–20]*.* Although mothers with a diagnosis of gestational diabetes mellitus (GDM) are more likely to have LGA infants [21, 22], maternal glucose concentrations within the normal range are associated with neonatal adiposity [21]. Studies comparing metabolism in obese and lean pregnant women have yielded inconclusive results for insulin sensitivity [20, 23, 24], which likely reflect small sample sizes, differences in study design [20] and confounding by co-existing disease [24].

The metabolic response to pregnancy evolves throughout gestation [16, 17], and longitudinal measurements are important. No previous studies have used the hyperinsulinaemic–euglycaemic clamp (HEC), to compare insulin sensitivity of glucose and lipid metabolism in such pregnant women. We aimed to compare metabolic responses to pregnancy in lean and very severely obese women using a large prospective longitudinal cohort, sampled at three time points in pregnancy and four months’ postpartum, to investigate differences in adiposity, glucose and lipid metabolism. At delivery, birthweights and complications were recorded.

Case–control studies were undertaken including: (1) in-depth metabolic assessments of insulin resistance at key time points, including whole-body glucose disposal, endogenous glucose production (EGP) and glycerol turnover using stable isotope methodology; (2) quantification of fat mass (FM) by MRI and magnetic resonance spectroscopy (MRS).

We aimed to examine women with normal glucose tolerance, to exclude the confounding effect of GDM.

## Methods

### Part I: longitudinal cohort study

#### Participants

**Fig. 1:**
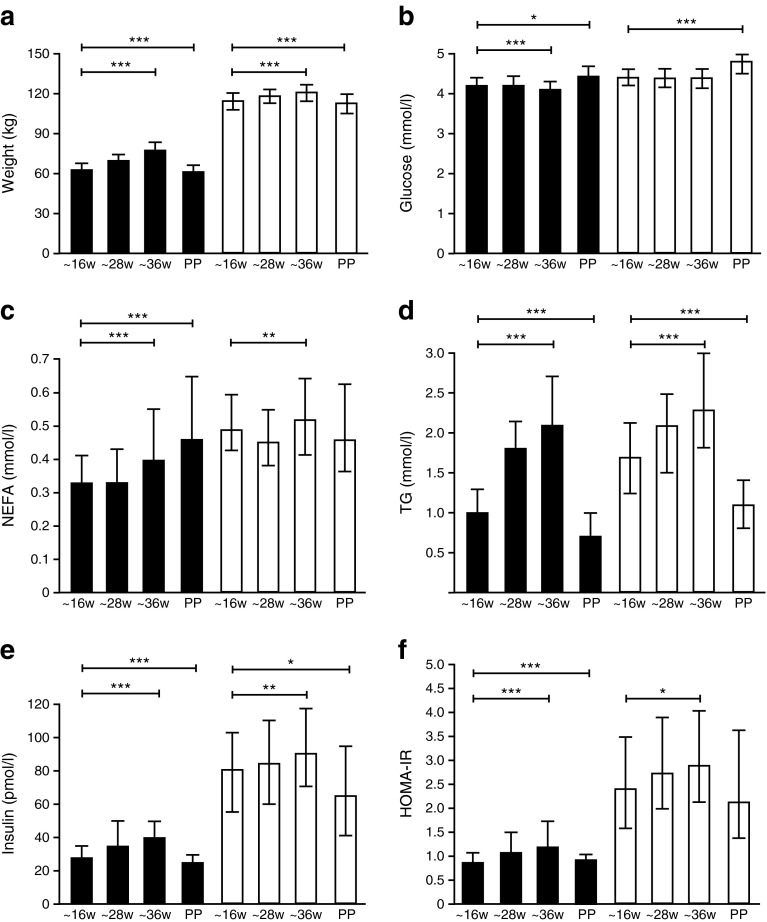
Weight and metabolic data of NGT obese and lean control participants. Data at early (~16 weeks [w]) (*n* = 190, obese [white bars], *n* = 118 lean [black bars]), mid (~28 weeks) and late (~36 weeks) gestation and postpartum. (**a**) Weight, (**b**) glucose, (**c**) NEFA, (**d**) TG, (**e**) insulin and (**f**) HOMA-IR. Median ± IQR demonstrated at each visit. Repeated ANOVA analyses were performed from the three visits during pregnancy and again on the four visits including the postpartum (PP) visit. *p < 0.05, **p < 0.01, ***p < 0.001

Participants were enrolled from May 2008 until May 2012 (Electronic Supplementary Material [ESM] Fig. 1). We recruited very severely obese women (booking BMI ≥40 kg/m^2^) from our antenatal clinic and lean (BMI ≥20 < 25 kg/m^2^) controls via referrals from the community to our clinic. Personal information (parity, ethnicity, smoking status, and social and demographic data) was collected [25]. The main exclusions were pre-existing diabetes, miscarriage prior to first assessment and major anomalies. Women who developed GDM (International Association of Diabetes in Pregnancy Study Guidelines [IADPSG] criteria) [26], or did not undergo an OGTT at 24–28 weeks’ gestation, or developed pre-eclampsia [27] or delivered <37 weeks’ gestation were excluded from analyses (ESM Fig. 1). Postnatal data were included regardless of breastfeeding status, hormonal contraception or stage of menstrual cycle.

#### Timing of assessments

We aimed to assess early (~16 weeks), mid (~28 weeks) and late (~36 weeks) gestation and postpartum (~4 months postpartum). Gestational age was calculated from the first day of the mother’s last menstrual period, or, from the dating scan. Actual gestational and post-delivery ages, median (interquartile range [IQR]), were early: 17.8 (16.1–19.4) weeks, mid: 28.1 (27.4–28.5) weeks and late: 36.1 (35.7–36.7) weeks, and 18.0 (15.0–22.5) weeks postpartum.

#### Anthropometric measurements

Women were weighed (SECA chair scales model 959, Birmingham, UK), their heights recorded, body fat estimated by bio-electrical impedance (Tanita Scales BC 420 MA, Amsterdam, the Netherlands) and BP recorded (Trimline Medical Products, Branchburg, NJ, USA).

#### Blood sampling

Venous blood samples at the above time points were collected after an overnight fast. A 2 h 75 g OGTT was performed between 24 and 28 weeks’ gestation and samples stored at −80°C for later determination of metabolites.

#### Laboratory analyses

Glucose concentrations were measured by a hexokinase method (Randox Laboratories, Co. Antrim, UK). NEFA, total cholesterol, HDL-cholesterol, triacylglycerol (TG) and liver function tests (LFTs) were measured by colorimetric methods (NEFA: Wako Chemicals, Neuss, Germany; total cholesterol: Olympus Diagnostics, Watford, UK; TG: Alpha Laboratories, Eastleigh, UK; LFTs: Abbott Diagnostics, Maidenhead, UK). Serum samples for insulin and C-peptide were analysed by ELISA (Mercodia, Uppsala, Sweden).

#### Assessment of insulin secretion and insulin resistance

HOMA was used to compute insulin secretion (HOMA%B) and insulin resistance (HOMA-IR) [28].

#### Obstetric outcome

Birthweight (SECA 384 scales, Birmingham, UK), sex and gestational age at delivery were recorded. An LGA infant was defined as a sex-specific birthweight for gestational age >90th percentile and an SGA infant <10th percentile [29]. Adverse obstetric outcomes recorded were the rate of Caesarean section, perineal tears and episiotomies during spontaneous vaginal deliveries, neonatal complications, maternal haemorrhage and admissions to the high dependency unit (HDU).

### Part II: case–control studies

#### Participants

In the longitudinal study, initial data revealed a decrease in circulating glucose in lean women only from early to late pregnancy (Fig. 1). To explore this, further groups of women were recruited in nested case–control studies, including: (1) lean and very severely obese pregnant and non-pregnant women, who underwent HEC studies with stable isotope tracer infusions at early (~19 weeks) or late (~36 weeks) gestation or when not pregnant; and (2) groups of lean and obese pregnant women who underwent MRI/MRS analysis of subcutaneous, visceral, intramuscular and intrahepatic fat at late gestation (Table 1). Exclusions were as in the longitudinal cohort study.

**Table 1:** Anthropometric and personal data of participants in all study groups

Variable	Case–control metabolic study				Case–control MRI/MRS study		*p* values for one-way ANOVA of four study groups	
	Pregnant lean	Pregnant obese	Non-pregnant lean	Non-pregnant obese	Pregnant lean	Pregnant obese	Lean	Obese
*n*	6	9	7	7	10	10		
Gestation at recruitment (days)	133 (130–140)	133 (129–139)	N/A	N/A	258 (254–261)	256 (252–258)		
Age (years)	35 ± 0.7^a,b^	27 ± 1.2***	25.9 ± 0.7	34.4 ± 1.9	32.5 ± 1.2	28.4 ± 1.1*	<0.0001	0.02
BMI (kg/m^2^)	24.2 (22.6–24.5)	41.2*** (38.8–44.8)	22.2 (21.3–23.7)	41.8 (36.6–45.2)	26.3 (24.9–28.8)	45.6*** (42.9–49.1)	0.46	0.33
Weight (kg)	63.6 (60.3–67.1)	115.6*** (113.0–124.0)	60.4 (57.9–65.6)	111.4 (96.4–116.8)	74.2 (65.8–81.2)	118.7*** (112.1–135.7)	0.84	0.48
Height (m)	1.65 (1.60–1.67)	1.68 (1.60–1.72)	1.66 (1.63–1.67)	1.63 (1.59–1.68)	1.67 (1.60–1.69)	1.64 (1.60–1.66)	0.18	0.54
Waist (cm)	85^a^ (80–98)	116*** (113–119)	74 (71–76)	115 (112–124)	N/A	N/A	0.003	0.99
Parity (0/1/>2; %)	17/83/0^a^	45/55/0	100/0/0^c^	20/40/60	60/30/10	30/40/30	0.005	0.40
Ethnicity (n[%]white)	6 (100)	9 (100)	7 (100)	7 (100)	10 (100)	10 (100)	0.81	0.67
Current smoker (%)	0	0	0	14	0	0	0.90	0.51
Social class (DEPCAT code)	2 ± 0	4 ± 0**	3 ± 1	4 ± 1	3 ± 0	4 ± 0*	0.14	0.95

Data are means ± SEM or median (IQR)

One-way ANOVA analyses were performed separately in lean control participants and obese participants from the four study groups (waist circumference in three study groups), which included (1) the longitudinal study (data not shown above—anthropometry demonstrated in Table 2); ethnicity 96% white in lean and obese groups; current smokers: 2% vs 11%** and DEPCAT code 3 ± 1 vs 4 ± 1*** in lean and obese groups; (2) the case–control study with pregnant participants and (3) case–control study with non-pregnant participants, and (4) MRI study with pregnant participants studied in late gestation only. Post hoc testing was carried out between the obese and lean control participants within the study groups and differences denoted:

*p < 0.05; **p < 0.01; ***p < 0.001

Post hoc testing was done between the obese participants in all the studies and the lean control participants in all the studies:

^a^Pregnant case–control study vs non-pregnant

^b^Pregnant case–control metabolic study vs pregnant case–control MRI study

^c^Non-pregnant vs pregnant case–control MRI study

There were no statistical differences between the following groups:

Pregnant longitudinal study vs pregnant case–control metabolic study

Pregnant longitudinal study vs non-pregnant

Pregnant longitudinal study vs pregnant case–control MRI study

DEPCAT code, deprivation category score (greater score indicates greater economic deprivation)

Anthropometric and laboratory analyses were performed as previously described.

#### HEC and tracer studies

Participants (*n* = 6–9/group at 19 weeks’ gestation) attended the Research Facility at 08:00 hours after an overnight fast [30]. Participants received infusions of [^2^H_2_]glucose and [^2^H_5_]glycerol (Cambridge Isotope Laboratories, Andover, MA, USA; prime 25 μmol/kg, continuous infusion 0.22 μmol kg^−1^ h^−1^ and prime 1.6 μmol/kg, continuous infusion 6.6 μmol kg^−1^ h^−1^, respectively) for 4.5 h. Blood samples were taken at 10 min intervals at the end of three 90 min periods with infusion of: (1) no insulin; (2) insulin (Actrapid, NovoNordisk, Bagsvaerd, Denmark.) 20 mU m^−2^ min^−1^ (suppresses lipolysis and EGP); and (3) insulin 40 mU m^−2^ min^−1^ (stimulates glucose uptake). Glucose was maintained at 5 mmol/l with 20% dextrose. Blood samples were centrifuged and plasma stored at −80°C. The quantity of glucose infused during the final 30 min of the high-dose HEC divided by the insulin concentrations was the insulin-stimulated glucose disposal index (*M*/I value) [31].

The rate of appearance and disappearance (Ra/Rd) of the tracee was calculated [31]:

$$ Ra=Rd=\left(F/TTR\kern0.5em \mathrm{plasma}\ \mathrm{tracer}\hbox{--}\ F\right) $$

where *F* is the infusion rate of tracer and TTR is the tracer:tracee ratio.

#### Mass spectrometry analyses

Standard curves were prepared [15] and glycerol triacetate *m/z* 217, [^2^H_5_]glycerol triacetate *m/z* 222, butanetriol triacetate *m/z* 231 (internal standard), glucose pentaacetate *m/z* 287, [^2^H_2_]glucose pentaacetate *m/z* 289 and [^13^C_6_]glucose pentaacetate *m/z* 293 (internal standard) monitored.

#### MRI

Whole-body MRI and proton MRS (^1^H-MRS) studies were performed on a 3T MAGNETOM Verio MRI system (Siemens AG Healthcare Sector, Erlangen, Germany). Women attended after an overnight fast, were positioned in the magnet on their left lateral side, and data from abdomen, liver, quadriceps and paraspinal tissue was acquired using a combination of spine and body matrix coil elements.

For ^1^H-MRS measurement of intramyocellular lipid (IMCL), single voxel (2 cm^3^) spectra localised to the right quadriceps muscle were acquired using a PRESS sequence (TR 5,000 ms/TE 30 ms) [32]. The hepatic fat fraction was derived using the Dixon method [14, 33]. Abdominal volume was estimated from 20 × 2 mm slices above the left renal pelvis.

To derive FM, adipose tissue volumes were analysed in triplicate using SliceOmatic v4 (TomOvision, Magog, QC, Canada). ^1^H-MRS analyses used the AMARES algorithm in jMRUI software version 3 (www.jmrui.eu/) [34, 35].

#### Sample size calculations and statistical analyses

Statistical analyses were performed (Stata 12, Stata Corporation, TX, USA). Data are presented as the mean ± SEM, or median (IQR) as appropriate. Categorical variables were compared using the *χ*^2^ or Fisher exact test. In the longitudinal cohort, the independent effects of lean vs very severely obese status and gestational age and their interaction were analysed using two-way repeated ANOVA. Post hoc analyses determined differences between lean vs very severely obese groups and differences between time points in the obese and then separately in the lean groups. Birthweight percentiles were adjusted for maternal age, socioeconomic status, smoking status and ethnicity in multiple logistic regression models to further explore differences between the groups. Logistic regression analyses were performed to determine if there was a metabolic indicator independent of obesity, associated with adverse obstetric outcomes.

In case–control metabolic studies, the independent effects of lean vs obese and gestational age/non-pregnant status and their interaction were analysed similarly with post hoc analyses (lean vs obese then gestational age/non-pregnant status in each group). For tracer studies two-way ANOVA analyses at basal state, low and high-dose insulin infusion were performed. MRI studies were compared by unpaired *t* tests or Mann–Whitney tests. A *p* value <0.05 indicated statistical significance.

We aimed to generate data for >200 obese and 100 lean women for the longitudinal study; formal sample size calculations were not performed. For the case–control study we calculated that the sample size used would have 80% power at the 5% significance level to show a difference between *M*/I values in obese and lean women that might be maintained by the end of pregnancy. The median (IQR) *M*/I value at term was 0.19 (0.11–0.26) in lean and 0.10 (0.08–0.21) in the obese groups. Therefore *n* = 30 per group would give 80% power to show these differences were significant at the 5% significance level.

#### Ethics approval

Participants provided written informed consent and the study was approved by institutional review boards (Lothian Research Ethics Committee, UK; reference 08/S1101/39) in accordance with principles endorsed by the Declaration of Helsinki.

## Results

### Part I: longitudinal cohort study

#### Participants

Women with very severe obesity (411) and lean women (138) at ~16 weeks’ gestation were assessed and following exclusions, data on 190 very severely obese and 118 lean control women were analysed (ESM Fig. 1). Postpartum declines and 3 month postnatal milestone not reached by termination of the study, explained lower numbers at this stage. Personal and anthropometric data are shown (Tables 1 and 2). Pregnant women in very severely obese groups were younger and more socioeconomically deprived than all lean pregnant groups (*p* < 0.05).

**Table 2:** Anthropometry and BP in longitudinal cohort study participants at early, mid and late gestation, and postpartum

Variable	Early gestation (~16 weeks)		Mid gestation (~28 weeks)		Late gestation (~36 weeks)		Postpartum (~3–6 months)	
	Lean	Obese	Lean	Obese	Lean	Obese	Lean	Obese
*n*	118	190	117	184	115	178	92	95
Gestation (days)	117 (106–126)	132*** (119–145)	199 (197–201)	194*** (191–199)	255 (253–257)	252*** (249–256)	122 (109–147)	130 (109–175)
BMI (kg/m^2^)	22.7 (21.4–23.8)	42.7*** (40.8–44.8)	25.0^a^ (23.8–26.3)	44.0***^a^ (42.1–46.4)	26.2^b,c^ (24.8–27.9)	45.0***^b,c^ (43.0–47.5)	22.5^d,e^ (21.3–24.1)	42.9***^d,e^ (40.3–46.0)
Weight (kg)	63.2 (57.7–67.3)	115.0*** (108.9–122.1)	70.0^a^ (64.4–74.2)	118.5***^a^ (112.3–124.4)	80.0^b,c^ (67.6–77.7)	121.0***^b,c^ (114.4–127.8)	61.4^d,e^ (58.3–67.6)	112.7***^d,e^ (106.6–122.2)
Body fat (%)	28.7 (25.7–32.7)	48.8*** (47.2–51.3)	32.5^a^ (29.1–35.7)	49.5*** (47.0–51.2)	34.7^b,c^ (30.3–37.4)	48.9*** (46.6–51.3)	28.9^d,e^ (24.8–33.1)	49.5*** (47.1–51.2)
Weight change (from 16 weeks)			6.2 (4.6–7.8)	2.9*** (0.7–5.3)	9.5^c^ (7.6–12.0)	5.4*** (2.9–9.4)	−0.7^d,e^ (−3.1–2.6)	0.3 (−4.4–3.5)
SBP (mmHg)	105 (100–110)	118*** (110–124)	108 (100–113)	118*** (110–120)	110^b,c^ (104–118)	120***^b,c^ (110–130)	108^e^ (100–112)	120***^e^ (110–125)
DBP (mmHg)	60 (60–67)	70*** (65–75)	60 (60–68)	70*** (66–74)	65^b,c^ (60–70)	72***^b,c^ (70–80)	65 (60–70)	70***^d,ef^ (68–80)

Data presented as median (IQR)

The independent effects of lean control vs obese status and gestational age, and their interaction, were examined using two-way repeated measures ANOVA on the variables in early, mid and late gestation in all the pregnant mothers and again in early, mid and late gestation and postpartum in the mothers attending the postpartum visit. All two-way repeated measures ANOVA analyses during pregnancy (*n* = 111 lean, *n* = 150 obese) and then analyses for all women attending postpartum (*n* = 85 lean, *n* = 85 obese) revealed significant differences in lean vs obese status, gestational age and significant interactions between these two variables (all *p* ≤ 0.01). The exception was the interaction for SBP and DBP in pregnancy and for the pregnancy + postpartum visit (both *p* > 0.05) (data not displayed)

Post hoc analyses for differences between lean controls vs obese at each time point were performed and differences denoted:

***p < 0.001

Post hoc analyses for differences between time points were done separately in the controls and obese and are denoted:

^a^Mid vs early gestation

^b^Late vs early gestation

^c^Late vs mid gestation

^d^Postpartum vs mid gestation

^e^Postpartum vs late gestation

^f^Postpartum vs early gestation

DBP, diastolic BP; SBP, systolic BP

#### Anthropometric and metabolic indices

Body weight and FM were greater in the very severely obese women and increased during pregnancy in both groups, although absolute weight gain was greater in lean controls at ~28 and ~36 weeks’ gestation (Fig. 1; Table 2). Percentage body fat increased during pregnancy in lean women only (Table 2). Systolic and diastolic BPs were greater in very severely obese compared with lean women (Table 2) and increased with advancing gestation in both groups.

Fasting glucose and insulin concentrations were higher in the very severely obese compared with the lean women at all visits (Fig. 1; Table 3). In both groups, glucose was lower and insulin concentrations higher during pregnancy than postpartum, with a progressive rise in insulin concentrations between 16 and 36 weeks of pregnancy. There were no differences in 2 h glucose concentrations at 24–28 weeks’ gestation (Table 3). HOMA%B and HOMA-IR were greater in very severely obese compared with lean women at all stages of pregnancy (Fig. 1; Table 3). However, in contrast to lean women, who displayed increasing HOMA-IR through pregnancy with a decrease postpartum, in the obese group there was no difference between mid and late gestation HOMA-IR or between postpartum and early pregnancy HOMA-IR. Similarly, TG, NEFA, total and LDL-cholesterol were greater at late compared with early gestation in each of obese and lean groups, with no difference in TG concentrations in obese compared with lean groups by late gestation (Table 3).

**Fig. 2:**
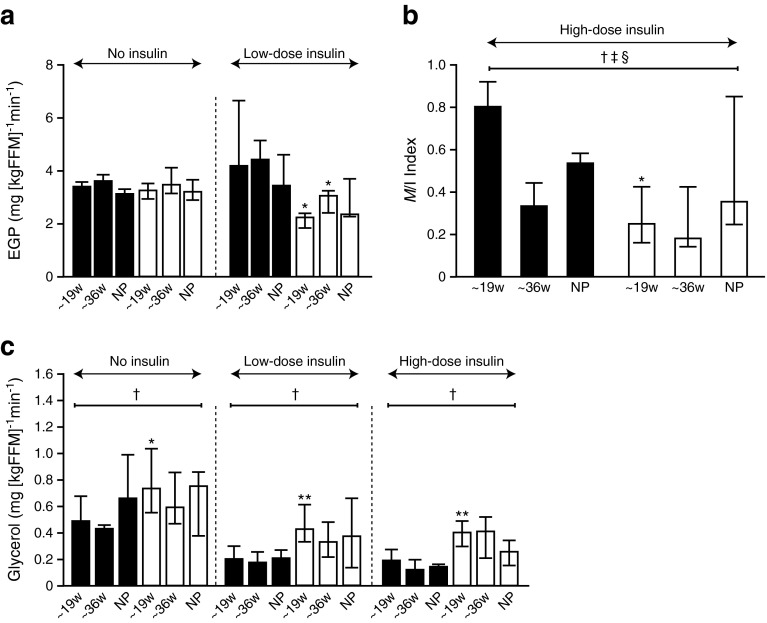
Infusion studies in obese and lean control pregnant and non-pregnant participants. (**a**) EGP (mg [kgFFM]^−1^ min^−1^) at baseline and low-dose insulin infusion; (**b**) *M*/I index is the stimulated glucose disposal rates with high-dose insulin infusion (mg [kgFFM]^−1^ min^−1^ divided by insulin concentrations at steady state [pmol/l × 10]); (**c**) glycerol turnover (mg [kgFFM]^−1^ min^−1^) at baseline, low-dose and high-dose insulin infusions. Two-way ANOVA analyses were performed on the variables to determine the independent effects of BMI and advancing gestation/non-pregnant state and their interaction. Obese (white bars); lean control (black bars); *p < 0.05, **p < 0.01, difference in obese vs lean at 19 weeks [w], 36 weeks and non-pregnant (NP). ^†^ *p* < 0.05 by two-way ANOVA for obese vs lean; ^‡^ *p* < 0.05 by two-way ANOVA for gestational age vs non-pregnant status; ^§^ *p* < 0.05 for two-way ANOVA interaction between obese vs lean and gestational age vs non-pregnant status. See ESM Figs 2 and 3 and ESM Table 2

**Table 3:** Metabolic data of longitudinal cohort study participants at early, mid and late gestation, and postpartum

Variable	Early gestation (~16 weeks)		Mid gestation (~28 weeks)		Late gestation (~36 weeks)		Postpartum (~3–6 months)	
	Lean	Obese	Lean	Obese	Lean	Obese	Lean	Obese
*n*	118	190	117	184	115	178	92	95
Gestation (days)	117 (106–126)	132* (119–145)	199 (197–201)	194* (191–199)	255 (253–257)	252* (249–256)	122 (109–147)	130 (109–175)
Fasting plasma glucose (mmol/l)	4.2 (4.0–4.4)	4.4* (4.2–4.6)	4.2 (4.0–4.5)	4.4* (4.2–4.7)	4.1 (3.9–4.3)	4.4*^a^ (4.1–4.6)	4.4^b,c,d^ (4.1–4.7)	4.8*^b,c,d^ (4.5–5.0)
2 h glucose (mmol/l)			4.9 (4.2–5.7)	5.3 (4.7–6.0)				
NEFA (mmol/l)	0.33 (0.25–0.42)	0.49* (0.42–0.59)	0.33 (0.24–0.44)	0.45*^e^ (0.38–0.55)	0.40^a,f^ (0.30–0.55)	0.52* (0.41–0.64)	0.46^b,c,d^ (0.37–0.65)	0.46 (0.36–0.63)
Total cholesterol (mmol/l)	4.9 (4.6–5.8)	5.2 (4.5–6.0)	6.4^e^ (5.4–7.4)	5.7*^e^ (5.0–6.8)	6.9^a,f^ (6.0–7.9)	6.1*^f^ (5.1–7.2)	5.1^c,d^ (4.6–6.1)	4.8*^b,c,d^ (4.0–5.4)
HDL-cholesterol (mmol/l)	1.9 (1.7–2.3)	1.5* (1.3–1.8)	1.9 (1.7–2.2)	1.6* (1.4–1.9)	1.8^f^ (1.5–2.1)	1.5* (1.3–1.8)	1.8^b,c^ (1.5–2.0)	1.4*^b,c^ (1.1–1.6)
LDL-cholesterol (mmol/l)	2.6 (2.0–3.4)	2.9* (2.4–3.5)	3.6^e^ (2.8–4.7)	3.3*^e^ (2.7–4.0)	4.1^a,b,f^ (3.4–5.1)	3.5*^f^ (2.7–4.1)	2.8^b,c,d^ (2.4–3.6)	2.9^c,d^ (2.2–3.5)
TG (mmol/l)	1.0 (0.9–1.3)	1.7* (1.3–2.2)	1.8^e^ (1.3–2.2)	2.1*^e^ (1.5–2.5)	2.1^a,f^ (1.6–2.7)	2.3^a,f^ (1.8–3.0)	0.7^b,c,d^ (0.5–1.0)	1.1*^b,c,d^ (0.8–1.4)
Insulin (pmol/l)	27 (19–34)	80* (55–104)	34 (26–50)	84*^e^ (63–114)	39^f^ (28–53)	90*^f^ (71–119)	24^d^ (17–30)	65*^c,d^ (41–96)
HOMA%B	123 (86–192)	288* (200–449)	161 (113–256)	312*^e^ (225–523)	232^a,f^ (155–353)	361*^f^ (219–531)	90^d^ (63–132)	176*^b,c,d^ (111–239)
HOMA-IR	0.85 (0.59–1.08)	2.4* (1.8–3.5)	1.04^e^ (0.77–1.49)	2.71*^e^ (2.00–3.91)	1.18^a,f^ (0.82–1.75)	2.9*^f^ (2.23–4.05)	0.76^b,c,d^ (0.50–0.97)	2.01*^c,d^ (1.39–3.53)
GGT (U/l)	10 (8–12)	13* (10–17)	8 (7–9)	11*^e^ (8–15)	9^a^ (8–12)	10*^f^ (8–14)	12^c^ (10–14)	18*^b,c,d^ (14–24)
Alk P (U/l)	54 (45–61)	75* (65–85)	82^e^ (73–93)	100*^e^ (81–113)	154^a,f^ (132–192)	150^a,b^ (126–181)	85^b,d^ (71–100)	96^b,d^ (79–112)
ALT (U/l)	12 (10–16)	15 (10–22)	15 (11–20)	13 (10–19)	15^f,a^ (11–18)	12 (9–18)	18 (13–24)	25^b,c,d^ (16–32)

Data presented as median (IQR)

The independent effects of lean control vs obese status and gestational age, and their interaction were examined using two-way repeated measures ANOVA on the variables in early, mid and late gestation in pregnancy, and again in early, mid and late gestation and postpartum time points

All two-way repeated measures ANOVA analyses during pregnancy (*n* = 111 lean, *n* = 150 obese) and then analyses for all women attending postpartum (*n* = 85 lean, *n* = 85 obese) revealed significant differences in lean vs obese status, gestational age ± postpartum visit and significant interactions between these two variables (all *p* < 0.05). The exceptions were: fasting plasma glucose, no effect of gestation and no interaction with obese/lean status; ALT, no effect of lean/obese status or gestational age/gestation + postpartum visit; GGT, no effect of gestation; HDL-cholesterol, insulin and HOMA%B, no interaction with obese/lean status with gestation/+ postpartum visit (*p* > 0.05) (data not displayed above)

Post hoc analyses for differences between lean controls vs obese at the respective time points were performed and differences denoted:

*p < 0.05

Post hoc analyses for differences between time points were done separately in the controls and obese and are denoted:

^a^Late vs mid gestation

^b^Postpartum vs early gestation

^c^Postpartum vs mid gestation

^d^Postpartum vs late gestation

^e^Mid vs early gestation

^f^Late vs early gestation

Alk P, alkaline phosphatase

The majority of metabolic variables changed with advancing gestation except fasting plasma glucose and alanine transaminase (ALT; Table 3). The liver-specific enzyme γ-glutamyl transferase (GGT), which may be increased with the presence of fat in the liver, decreased in all mothers through pregnancy but this decrease started in mid-pregnancy in the obese and later in the lean mothers.

#### Obstetric outcomes

Birthweights from very severely obese vs lean mothers were greater: 3,636 ± 37 vs 3,556 ± 43 g (*p* = 0.04) with, after adjusting for maternal factors, an increased prevalence of LGA infants: 41% vs 31%, respectively (*p* = 0.04; ESM Table 1) but no difference in prevalence of SGA infants. Preterm births in the obese group were greater (ESM Fig. 1). In the lean vs very severely obese women, the Caesarean section rate was 19% vs 42% (*p* < 0.001); 55% and 46% were emergency sections in the groups, respectively (*p* = 0.06). In those delivering by spontaneous vaginal delivery, instrumentation was necessary in 46% vs 41% and perineal tears/episiotomy was observed/performed in 78% vs 76% of lean vs very severely obese mothers (both *p* > 0.05). Postpartum haemorrhage was 1% vs 5%, maternal HDU admissions 5% vs 12.5% and neonatal HDU admissions 5% vs 4% in lean vs very severely obese mothers, respectively (all *p* > 0.05). Fasting glucose concentrations in the third trimester was the only metabolite significantly associated with any adverse obstetric outcome but there were no metabolic associations with Caesarean section alone.

### Part II: case–control study

#### Participants

In case–control studies, metabolism was explored with HEC/metabolic tracer infusions and fat distribution determined with MRI/MRS studies (Table 1). Exclusions and withdrawals were only relevant in the infusion studies at 36 weeks’ gestation where one lean and three very severely obese women were excluded after a diagnosis of GDM, and one lean and one very severely obese mother withdrew. In the tracer studies, women diagnosed with GDM had data from their 19 week clamp included in analyses. Participants in these case–control studies were representative of those in the longitudinal cohort study. Personal and anthropometric data are shown (Tables 1 and 2).

#### Metabolic tracer and HEC studies

The technical success of the HECs are shown (ESM Figs 2 and 3; ESM Table 2). At 19 weeks’ gestation, basal glucose and insulin concentrations (i.e. no insulin infusion) were greater in very severely obese vs lean women but were comparable otherwise (ESM Table 2). In the absence of insulin, EGP (derived from [^2^H_2_]glucose infusion) did not change during pregnancy in either lean or very severely obese women, and was not different in pregnancy compared with non-pregnant controls (Fig. 2). During low-dose insulin infusion, EGP was variable in non-pregnant women, and similar between groups; in pregnancy, EGP was suppressed in very severely obese but not lean women at 19 and 36 weeks’ gestation (Fig. 2), suggesting greater hepatic insulin sensitivity during pregnancy in obese women. During the high-dose HECs, insulin-stimulated glucose disposal (*M*/I) showed a marked diminution from early to late pregnancy in the lean group. Glucose disposal was lower in very severely obese vs lean women at 19 weeks’ gestation only with similar insulin-stimulated glucose disposal by 36 weeks’ gestation (Fig. 2).

Lipolysis (quantified with [^2^H_5_]glycerol infusion) was suppressed by insulin infusion in each of the non-pregnant and pregnant women (Fig. 2). Lipolysis was greater in obese compared with lean women at 19 weeks’ gestation in the basal state and during the low- and high-dose HEC. At 36 weeks’ gestation there were no differences between lean and obese groups. There were no associations between body fat distribution and glycerol turnover in early and late pregnancy and in the non-pregnant state in lean and obese women.

#### Body fat distribution

**Fig. 3:**
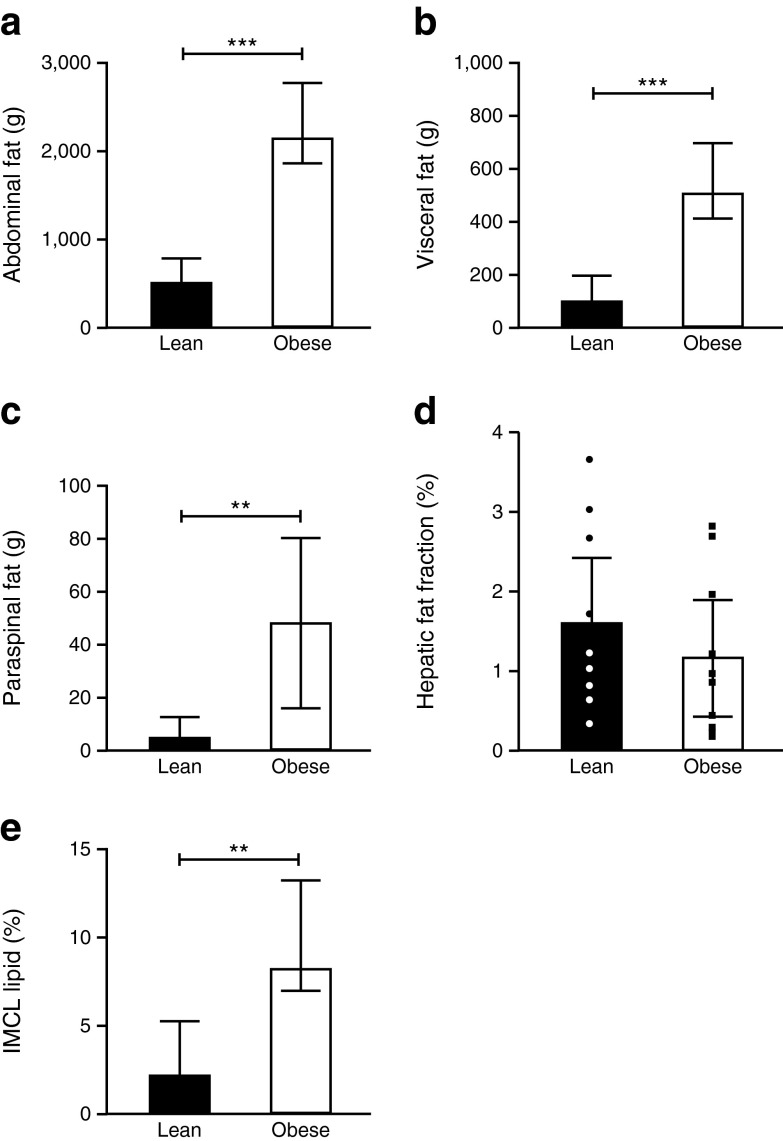
MRI studies in obese and lean control pregnant participants at 36 weeks’ gestation. FM in (**a**) abdominal subcutaneous depot, (**b**) intra-abdominal, (**c**) paraspinal, (**d**) hepatic fat fraction (%) and (**e**) IMCL (quadriceps) as estimated by MRI at 36 weeks’ gestation in *n* = 10 obese participants (white bars) and *n* = 10 lean controls (black bars). For the hepatic fat fraction, individual participants’ data points are shown (lean, black and white circles; obese, black squares). Data compared between groups using unpaired *t* tests or Mann–Whitney tests as appropriate; **p < 0.01; ***p < 0.001

Subcutaneous abdominal, intra-abdominal visceral, paraspinal FM depots and IMCL were greater but there was no difference in hepatic fat at 36 weeks’ gestation in the obese vs lean women, respectively (Fig. 3).

## Discussion

We hypothesised that the combination of very severe obesity and pregnancy would lead to exaggeration of the normal, pregnancy-related insulin resistance at all stages of pregnancy and anticipated greater circulating glucose, TG and NEFA concentrations in these women compared with gestation-matched lean controls.

In our longitudinal cohort study, circulating glucose and NEFA concentrations were higher in the very severely obese women throughout pregnancy. However, fasting glucose did not rise during pregnancy and although a rise in HOMA-IR was seen in early- to mid-pregnancy, there was no further increase in HOMA-IR by late pregnancy in obese women. The difference between lean and the very severely obese women observed in early pregnancy with respect to TG levels was attenuated by late gestation.

This pattern was confirmed in metabolic studies using stable isotope tracers with HECs, which showed that very severely obese women are resistant to insulin-mediated stimulation of glucose disposal and suppression of lipolysis throughout pregnancy, but in lean women there is a progressive decline in insulin sensitivity during pregnancy so that differences between obese and lean groups in mid-gestation were attenuated by late pregnancy. Previous studies demonstrate the importance of TG in obese pregnant women where the failure of insulin to suppress maternal lipolysis [19] leads to increased circulating TG which may be hydrolysed at the placenta, leading to increased fatty acids crossing the placenta hence contributing to fetal fuel excess and macrosomia [18]. In the very severely obese women studied this appears to be attenuated by term. Glycerol turnover was not associated with body fat distribution, in contrast to other studies which have shown significant associations with obesity [36]. Visceral adipocytes have greater lipolytic activity per kilogram FM, although upper subcutaneous adipose tissue contributes the greatest supply of NEFA [37] and differences in β-adrenoceptor density [38] and other receptors in adipose tissue [39], and sensitivity to hormones that regulate lipolysis [38], may be important [30, 40]. In very severe obesity, body habitus may be a less important factor compared with the obese state per se, although as the portal circulation is not directly sampled, associations between central obesity and glycerol turnover may be missed [41].

The differences during insulin infusion may reflect altered insulin sensitivity in skeletal muscle (glucose uptake) and adipose tissue (lipolysis). In the fasting low-insulin state, hepatic insulin sensitivity is a determinant of glucose homeostasis. Our clamp studies showed that insulin sensitivity in the liver improved during pregnancy in the very severely obese women but was unchanged in lean ones as reflected by the EGP. Despite convergence in metabolism by term between the two groups, the very severely obese pregnant women had infants with significantly greater birthweights. Nevertheless it may be that further increases in birthweight at late gestation—a period of time when there is accretion of FM in the baby—are ameliorated at this important time. The complications in the very severely obese pregnant women with greater rates of macrosomia and Caesarean section rates are in keeping with other studies [5, 6]. Contrary to some studies [18], we did not show a greater prevalence of SGA offspring from obese mothers perhaps because these mothers had normal glucose tolerance and were normotensive with a low prevalence of cigarette smoking. Glucose concentrations during pregnancy were a predictor of adverse obstetric outcome independent from obesity as previously reported [42], but were not associated with Caesarean section rates reflecting the importance of other factors in determining Caesarean sections. Our evidence that pregnancy has more profound metabolic effects on lean women concord with previous human [43] and rodent studies, where excess glucose intolerance in a high-fat-fed group in early pregnancy was not present by late pregnancy [44] as well as other animal studies [45].

Regarding insulin sensitivity during pregnancy, we considered whether analyses of fat depots was important in pregnancy [43]. Although our obese women had greater BMI and greater subcutaneous, visceral and paraspinal adipose depots and skeletal muscle at the end of pregnancy, there was no evidence of increased intrahepatic fat, agreeing with previous human studies using ultrasound [46]. The lack of association between intrahepatic fat with obesity in pregnancy is consistent with our animal model [44]. To our knowledge, this is the first study to quantify IMCL or intrahepatic fat in pregnancy. Liver fat levels measured were lower than one would expect in non-pregnant very severely obese women [13, 47, 48] but are concordant with similar convergence of circulating liver enzyme levels between the very severely obese and lean women towards the end of pregnancy, suggesting that pregnancy protects from liver fat accumulation and suggest a predilection for fatty acid oxidation from intrahepatic sources rather than from IMCL lipid, which may underpin the improved hepatic insulin sensitivity noted above, although confirmatory studies are required.

It may be that the hepatic fat in the very severely obese mothers is mobilised earlier as evidenced by the GGT concentrations starting to decrease in mid- as opposed to late gestation as was seen in the lean controls. Of note there was a significant rise in ALT by late gestation in the lean mothers only, which may reflect that gluconeogenesis [49] was upregulated in these women alone; ALT concentrations were lower in the pregnancies complicated by severe obesity compared with the non-pregnant state, whereas this was not the case in the lean women. These observations are consistent with a ‘metabolic adaptation’ during pregnancy in the very severely obese pregnant mothers and a more exaggerated response during pregnancy in the lean mothers.

Our in-depth data in a large longitudinal study provide insights into the metabolic changes during pregnancies complicated by obesity. Importantly, these changes reflect those in healthy obese pregnant women, and not those with GDM. In the case–control study, we excluded data from one lean and three obese participants after they developed GDM (according to the strict thresholds of the IADPSG criteria, [26]). The data from these women were still used at earlier time points. A limitation in the longitudinal study is the decrease in numbers followed up postpartum. However, uniquely in our study with the aid of two representative case–control study groups that included non-pregnant women, we have been able to understand the divergent changes in insulin sensitivity in the different body depots during pregnancy. Postpartum studies of the hepatic fat in these women would be of particular interest.

In summary, we demonstrate that, contrary to expectations, differences in metabolism between lean and very severely obese mothers during pregnancy converge by the end of pregnancy, with a more exaggerated metabolic response to pregnancy in lean than very severely obese women, chiming with the concept of ‘metabolic inflexibility’ in such obese individuals [50]. However, in mid-gestation, these obese women have substantial insulin resistance compared with lean women, which they have presumably exhibited since conception. This suggests that, if maternal metabolic abnormalities underlie adverse pregnancy outcomes, then therapeutic intervention needs to be provided early in pregnancy.

## Electronic supplementary material

ESM Fig. 1 (PDF 139 kb)

ESM Fig. 2 (PDF 422 kb)

ESM Fig. 3 (PDF 430 kb)

ESM Table 1 (PDF 220 kb)

ESM Table 2 (PDF 233 kb)

## Abbreviations

ALT: Alanine transaminase
EGP: Endogenous glucose production
FM: Fat mass
GDM: Gestational diabetes mellitus
HEC: Hyperinsulinaemic–euglycaemic clamp
HDU: High dependency unit
HOMA%B: HOMA-insulin secretion
IADPSG: International Association of Diabetes in Pregnancy Study Guidelines
IMCL: Intramyocellular lipid
IQR: Interquartile range
LGA: Large for gestational age
MRS: Magnetic resonance spectroscopy
SGA: Small for gestational age
TG: Triacylglycerol
