# Supplementary material for: Convergence in insulin resistance between very severely obese and lean women at the end of pregnancy
Source: Diabetologia. 2015 Aug 7;58(11):2615–26. doi: 10.1007/s00125-015-3708-3 (PMC4589551; doi:10.1007/s00125-015-3708-3)
Supplement: Supplementary file 1 — (PDF 139 kb) [file 125_2015_3708_MOESM1_ESM.pdf]

ESM Fig. 1 Participants

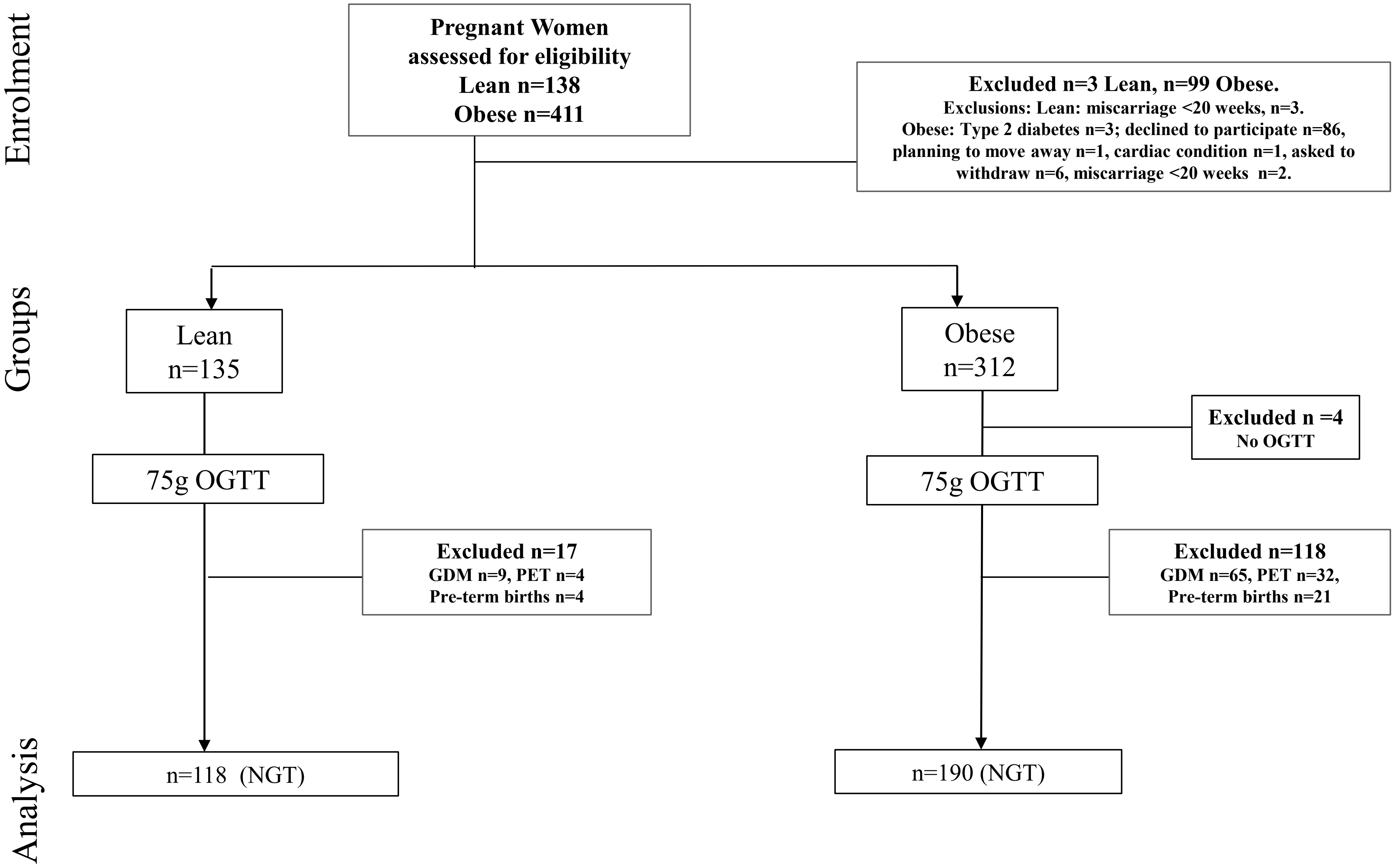

Lean control and Obese participants were recruited through the metabolic antenatal clinic. Those participants with normal glucose tolerance by IADPSG criteria with term infants >259 days gestation were analysed. OGTT: oral glucose tolerance test; NGT: normal glucose tolerance; PET: pre-eclampsia.
