# Supplementary material for: Convergence in insulin resistance between very severely obese and lean women at the end of pregnancy
Source: Diabetologia. 2015 Aug 7;58(11):2615–26. doi: 10.1007/s00125-015-3708-3 (PMC4589551; doi:10.1007/s00125-015-3708-3)
Supplement: Supplementary file 2 — (PDF 422 kb) [file 125_2015_3708_MOESM2_ESM.pdf]

## ESM Fig. 2 Glucose and insulin concentrations during infusion studies

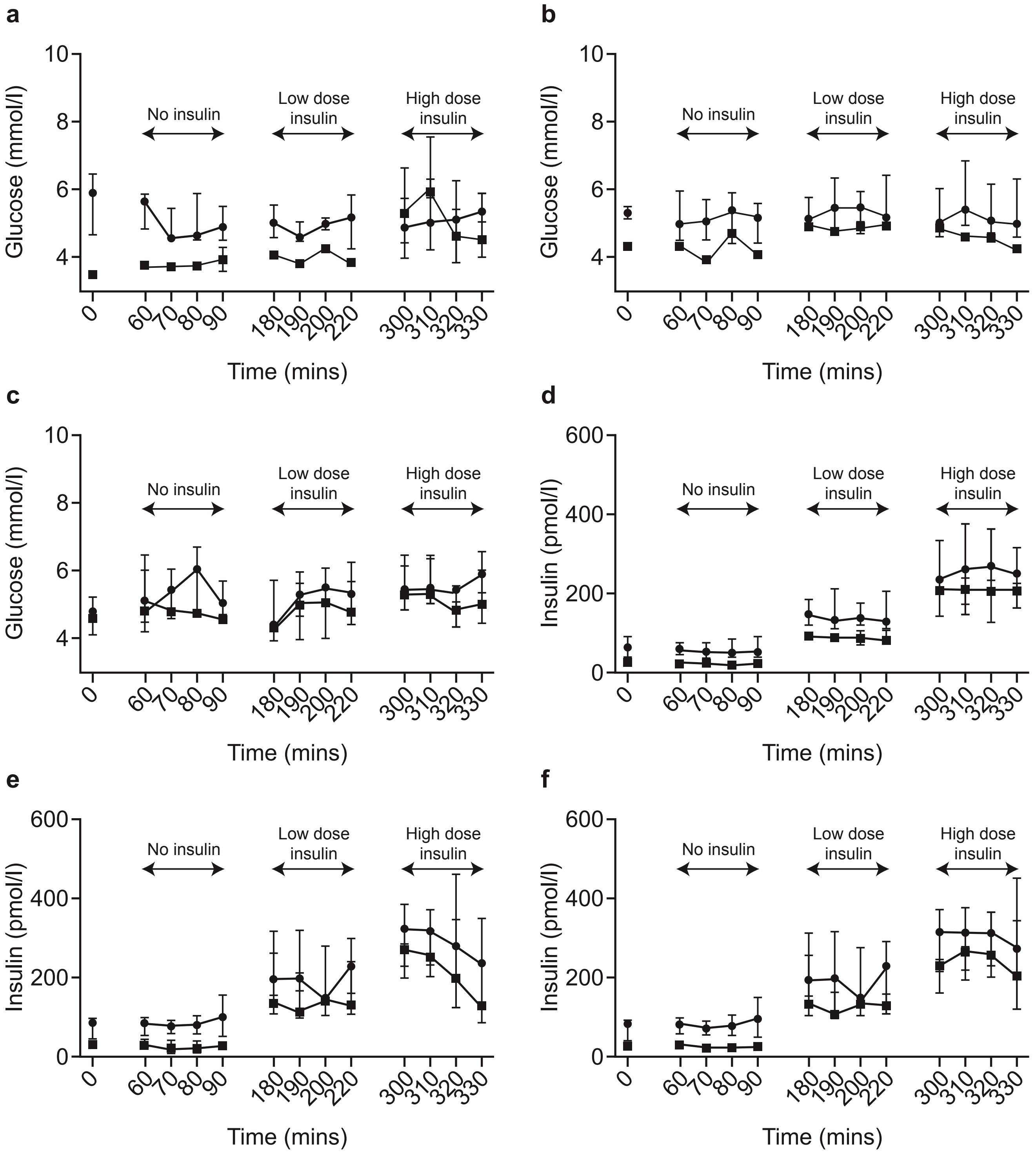


Glucose concentrations during the stable isotope infusion studies at baseline steady state (60-90 minutes), low-dose insulin infusion (180-210 minutes) and at high dose insulin infusion (300-330 minutes) are presented as median (IQR) at 19 weeks gestation (**a**), 36 weeks gestation (**b**) and in the non-pregnant state (**c**) and for insulin concentrations at 19 weeks gestation (**d**), 36 weeks gestation (**e**) and in the non-pregnant state (**f**). Obese subjects (black circles), Lean subjects (black squares). See ESM Table 2.
