# Supplementary material for: Convergence in insulin resistance between very severely obese and lean women at the end of pregnancy
Source: Diabetologia. 2015 Aug 7;58(11):2615–26. doi: 10.1007/s00125-015-3708-3 (PMC4589551; doi:10.1007/s00125-015-3708-3)
Supplement: Supplementary file 4 — (PDF 220 kb) [file 125_2015_3708_MOESM4_ESM.pdf]

ESM Table 1 Birth weights, centiles and metrics

	Control	Obese	p	p
N	**118**	**190**	**Unadjusted**	**Adjusted[1]**
Birth weight (g)	3556±43	3636±37	0.17	0.04
Birth weight >90th percentile(n(%))	37(31.3)	77(40.5)	0.14	0.04
Birth weight <10th percentile(n(%))	6(5.0)	10(5.3)	0.98	0.86
Gestational age (days)	283 (277-289)	283 (274-290)	0.60	
Male gender (n(%))	57(46.7)	94(47.2)	0.93	

Obstetric outcomes in very severely obese and control participants with normal glucose tolerance. Data presented as absolute numbers (%), mean±SEM or median(IQR) for skewed data. Birth weights between groups were compared after adjustment for maternal age, gestational age, parity, infant gender, smoking status, DEPCAT code and ethnicity[1]. Birth weight percentiles take into account parity, gestational age and gender of infant. Birth weight percentiles were further adjusted for maternal age, smoking status, DEPCAT code and ethnicity[1].
