# Supplementary material for: Convergence in insulin resistance between very severely obese and lean women at the end of pregnancy
Source: Diabetologia. 2015 Aug 7;58(11):2615–26. doi: 10.1007/s00125-015-3708-3 (PMC4589551; doi:10.1007/s00125-015-3708-3)
Supplement: Supplementary file 5 — (PDF 233 kb) [file 125_2015_3708_MOESM5_ESM.pdf]

ESM Table 2 Insulin infusion studies in pregnant and non-pregnant obese and lean control subjects

a) 19 weeks gestation

	No insulin infusion/Basal		Low dose insulin infusion		High dose insulin infusion		P values for 2 way ANOVA		
	**Lean**	**Obese**	**Lean**	**Obese**	**Lean**	**Obese**	**Lean vs. Obese**	**No insulin vs. insulin**	**Interaction**
Glucose (mmol/l)	4.2 (3.9- 4.3)	4.8 (4.7-5.1) *	4.6 (4.0-5.2)	4.8 (4.6-4.9)	5.1 (4.2-6.2)	5.1 (4.8-5.3)	0.03	0.17	0.34
Insulin (pmol/l)	18 (17 – 23)	52 (39 – 86) **	85 (73 – 106)	132 (15 – 209) *	206 (158 – 227)	250 (207 – 327) *	0.0007	<0.0001	0.61
Glucose (d2) Enrichment %	2.6 (2.5-3.0)	3.6 (3.2-3.9) **	1.9 (1.6-2.2)	3.5 (3.3-4.0) ***	1.0 (0.8-1.5)	2.4 (2.1-3.2) ***	0.002	<0.0001	0.36
Glycerol (d5) Enrichment %	2.8 (2.3 – 3.6)	2.5 (2.0 – 3.4)	6.9 (5.9 – 8.1)	4.3 (3.7 – 5.4)**	7.8 (6.0 – 9.1)	4.8 (4.0 – 6.0) *	0.0004	<0.0001	0.09

b) 36 weeks gestation

	No insulin infusion/Basal		Low dose insulin infusion		High dose insulin infusion		P values for 2 way ANOVA		
	**Lean**	**Obese**	**Lean**	**Obese**	**Lean**	**Obese**	**Lean vs. Obese**	**No insulin vs. insulin**	**Interaction**
Glucose (mmol/l)	3.8 (3.7-4.1)	5.0 (4.8-5.5)	4.4 (4.0- 4.8)	5.3 (5.1-8.0)	4.7 (4.4-5.1)	5.1 (4.7-5.8)	0.11	0.64	0.97
Insulin (pmol/l)	25 (23 – 42)	94 (59 – 107)	126 (113 – 246)	193 (156 – 304)	205 (189 – 362)	281 (204 – 352)	0.11	0.001	0.86
Glucose (d2) Enrichment %	2.7 (2.6-2.9)	3.4 (2.8-3.6)	2.1 (1.9-2.4)	3.2 (2.6- 3.5) *	1.5 (1.4-1.7)	2.6 (2.1-3.1) *	<0.0001	<0.0001	0.45
Glycerol (d5) Enrichment %	3.9 (3.1 – 4.9)	3.1 (2.3 – 3.6)	8.2 (7.2 – 9.6)	5.0 (4.8 – 7.7)	10.2 (9.7 – 10.4)	4.3 (4.3 – 8.1) *	0.003	0.0007	0.20

c) Non pregnant

	No insulin infusion/basal		Low dose insulin infusion		High dose insulin infusion		P values for 2 way ANOVA		
	**Lean**	**Obese**	**Lean**	**Obese**	**Lean**	**Obese**	**Lean vs. Obese**	**No insulin vs. insulin**	**Interaction**
Glucose (mmol/l)	4.5(4.4-5.6)	5.0(4.5-5.2)	4.7(4.6-5.1)	4.7(4.6-5.1)	5.2(4.7-5.1)	5.0(4.7-5.5)	0.21	0.33	0.92
Insulin (pmol/l)	24 (18-29)	45 (29 – 83)	123 (96 – 128)	139 (100 – 190)	281 (229 – 300)	243 (207 – 297)	0.34	<0.0001	0.52
Glucose (d2) Enrichment %	2.6 (2.4-2.8)	3.5 (3.3-3.6)	2.2 (1.9-2.3)	3.4 (2.6- 4.2)	1.06 (0.9 -1.3)	1.6 (1.1-2.7)	0.66	< 0.0001	< 0.0001
Glycerol (d5) Enrichment %	2.6 (2.4 – 2.8)	2.7 (2.5 – 4.0)	2.3 (2.0 – 2.4)	5.0 (4.1 – 9.5) ***	1.2 (1.0 – 1.2)	7.4 (6.1 – 9.2) ***	< 0.0001	0.14	0.004

Data presented as median (IQR). Steady state glucose and insulin concentrations, glucose (d2) enrichment and glycerol (d5) enrichment (moles % excess) during basal infusion studies (no insulin), low-dose and high-dose insulin infusion studies are shown. The independent effects of lean control vs. obese status and the effect of no insulin

vs. insulin infusion and their interaction, were examined using 2-way ANOVA analyses. Post-hoc testing was done between the obese and control participants within the study groups and differences denoted: *$p<0.05$; **$p<0.01$; ***$p<0.001$. The independent effect of gestational age/non-pregnant status on glucose concentrations was also examined using 2-way ANOVA analysis in turn at basal state and during low dose insulin infusion and high dose insulin infusion in the lean control group then in the obese group. There were no differences in glucose concentrations at 19 weeks gestation vs. 36 weeks gestation vs. the non-pregnant state when tested in turn in the basal state/no insulin, low dose insulin and then high dose insulin infusion studies ($p>0.05$) confirming the technical success of the clamp studies.
